# Retrospective analysis of pediatric sepsis and the burden of antimicrobial resistance in Duhok, Kurdistan Region of Iraq

**DOI:** 10.3389/fphar.2024.1347832

**Published:** 2024-02-26

**Authors:** Delveen R. Ibrahim, Abdulrhaman T. Saadi, Nizar B. Yahya, Marwa S. Ibrahim, Ali Y. Saeed, Sawsan S. Abdulaziz, Revan Y. Hasqyal, Berivan K. Alarsalani, Khalid S. Ibrahim

**Affiliations:** ^1^ Department of Biology, Collage of Science, University of Duhok, Duhok, Kurdistan Region, Iraq; ^2^ Medical Microbiology Department, College of Medicine, University of Duhok, Duhok, Kurdistan Region, Iraq; ^3^ Heevi Pediatrics Teaching Hospital, Duhok Health Directorate, Duhok, Kurdistan Region, Iraq; ^4^ Pediatrics Department, College of Medicine, University of Duhok, Duhok, Kurdistan Region, Iraq; ^5^ Department of Medical Laboratory Technology, College of Health and Medical Techniques-Shekhan, Duhok Polytechnic University, Duhok, Kurdistan Region, Iraq; ^6^ Department of Biology, College of Sciences, University of Zakho, Zakho, Kurdistan Region, Iraq

**Keywords:** sepsis, pediatric, retrospective, antimicrobial resistance, Duhok-Iraq

## Abstract

**Introduction:** Sepsis is a life-threatening complication in pediatric patients. This study primarily aimed to investigate sepsis-causing bacteria and their antimicrobial resistance profile and check the change in the antimicrobial resistance trend for some selected bacteria. In addition, we evaluated the incidence of sepsis, the related mortality rate, and the effectiveness and outcome of the treatment regimes in sepsis pediatric patients.

**Methods:** A retrospective analysis was conducted on 4-year data (2018–2021) collected from three intensive care units at the Hevi Pediatric Teaching Hospital. Sepsis screening involved clinical detection and confirmation by blood culture.

**Results:** A total of 520 out of 1,098 (47.35%) blood samples showed positive microbial growth. A decrease in sepsis rate was observed during the COVID-19 pandemic. Coagulase-negative Staphylococci (CoNS) and *Klebsiella pneumonia* were the most commonly isolated bacteria. A notable variation in the antimicrobial resistance trend was observed among sepsis-causing bacteria. The empirical sepsis treatment recommended by the WHO was ineffective, as certain bacteria exhibited 100% resistance to every antibiotic tested. The mortality rate significantly increased from 1.3% in 2018 to 16.5% in 2021.

**Discussion:** The antimicrobial resistance profile of sepsis causing bacteria is of concerns, indicating a potentially serious situation. Thus, to avoid treatment failure, the monitoring of antimicrobial resistance in pediatric patients is essential.

## 1 Introduction

Sepsis represents the systemic condition that arises from microbial infections associated with clinical findings and hemodynamic changes. It is a state of immune imbalance and an immune response to microbial invasion that results in organ injury that is sometimes associated with severe morbidity and mortality (Hotchkiss et al., 2016; Odabasi and Bulbul, 2020). In 2017, an estimated 48.9 million cases of sepsis were recorded worldwide and 11.0 million sepsis-related deaths were reported (Rudd et al., 2020). Sepsis remains a major health problem for critically ill adults and children. Additionally, it is one of the leading causes of death in infants and children (Jiang et al., 2020; Hermon et al., 2021). Neonatal sepsis is classified as early-onset (<72 h) and late-onset (4–30 days) according to the time of onset (WHO, 2022).

The excessive and improper use of antibiotics, particularly broad-spectrum antibiotics, plays a major role in the emergence of drug-resistant strains, as observed in several studies (Doern et al., 2014; Tessema et al., 2021; Hasan and Ibrahim, 2022; Hami and Ibrahim, 2023; Ibrahim, 2023). Neonatal sepsis caused by antimicrobial-resistant (AMR) bacteria is a multifactorial issue of global concern due to the increased illness, death, and hospital expenses associated with it. Recent research revealed that the antibiotic resistance patterns of the bacterial pathogens responsible for newborn sepsis vary between hospital settings and time periods, which might be due to the different treatment regimens used in neonatal care (Tessema et al., 2021).

The current study aimed to evaluate the prevalence and susceptibility of the predominant sepsis-causing bacteria among pediatric patients in the Hevi Pediatric Teaching Hospital, Kurdistan region of Iraq. This will provide an epidemiological analysis of sepsis data among patients. In addition, the correlation between the gender and age of patients and the rates of mortality within the studied population was analyzed. Accordingly, suggestions are made regarding sepsis identification and patient treatment regimens.

## 2 Materials and methods

### 2.1 Samples and data collection

The current study used data collected from the intensive care unit at the Hevi Pediatric Teaching Hospital in Duhok Governorate, Iraq. Four-year data were obtained from between 1 January 2018, and 31 December 2021. During the study period, 1,098 blood samples were collected from both genders from 3,865 patients. The samples were collected from suspected sepsis patients who were referred by pediatric consultants; the ages of the patient ranged from 1 day to 15 years, with 771 males and 327 females. The samples were processed in the microbiology laboratory at the same hospital. The 5-age pediatric patient subgroup’s demographic data were designated based on a previous study (Hermon et al., 2021) (Supplementary Table S1).

### 2.2 Inclusion and exclusion criteria

All clinically suspected cases of sepsis (newborns and children) from the age of 1 day–15 years admitted to the pediatric intensive care unit (PICU), neonatal intensive care unit (NICU), and semi intensive care unit (ICU) were included in the study. The criteria for clinically suspected sepsis were based on signs of infections. This involved fever, elevated heart rate, rapid breathing, or signs of a localized infection, such as a skin or urinary tract infection. Furthermore, some cases showed clinical deterioration as the child may exhibit signs of systemic illness, such as lethargy, irritability, poor feeding, or decreased responsiveness. In addition, abnormal vital signs were recorded, including abnormal body temperature (either fever or hypothermia), tachycardia, or tachypnoea. For all suspected sepsis patients, detailed laboratory tests were performed, e.g., complete blood picture (CBP), C-reactive protein (CRP), and blood cultures.

The exclusion criteria were based on two main factors: the age of the patient and sepsis symptoms. Any child cases that were over 15 years old or did not exhibit sepsis symptoms were not included in this research.

### 2.3 Ethics

The approval for conducting this study was provided by the Ethical Committee for Medical and General Health Research Ethics from the Duhok Directorate General of Health (14012018-0001).

### 2.4 Culture

The blood samples were immediately transferred to BACT/ALERT®PF PLUS pediatric culture bottles and placed in a BACT/ALERT 3D instrument for 2–5 days (De La Rica et al., 2016). A small amount of culture was inoculated on blood agar (BA), chocolate agar (CHOC), and MacConkey (MAC) (Neogen, UK) and incubated overnight up to 24 h in case bacterial growth was observed. Isolates were identified using classical methods (Mahon et al., 2014) and an automated system (Vitek2, BioMerieux, France).

### 2.5 Antibiotics susceptibility test

Various antibiotic discs (Bioanalyses, Turkey) were used for antimicrobial susceptibility tests (Supplementary Tables S4, S5). The tests were performed manually (CLSI, 2017) from 1 January 2018, to 19 May 2019. Mueller–Hinton agar (MHA) (Neogen, UK) was used for the disc diffusion test. The diameters of the inhibition zones around each disc were measured and compared with the standard inhibition zone, as recommended by the Clinical Laboratory Standard Institution (CLSI, 2017). The results were interpreted as sensitive or resistant; intermediate resistance was also considered as resistance. On the other hand, from 20 May 2019, onward, the antimicrobial susceptibility test was carried out using an automated system with a Vitek®2 AST-P640 card for Gram-positive bacteria (G+) and AST-N327 cards for Gram-negative bacteria (G−) (BioMerieux, France).

### 2.6 Statistical analysis

GraphPad Prism 8.1 software and Excel were used for statistical analysis. The Chi-square test and two-way ANOVA were used to compare significant differences between data. *p* < 0.05 was considered statistically significant.

## 3 Results

### 3.1 The prevalence of sepsis

**FIGURE 1:**
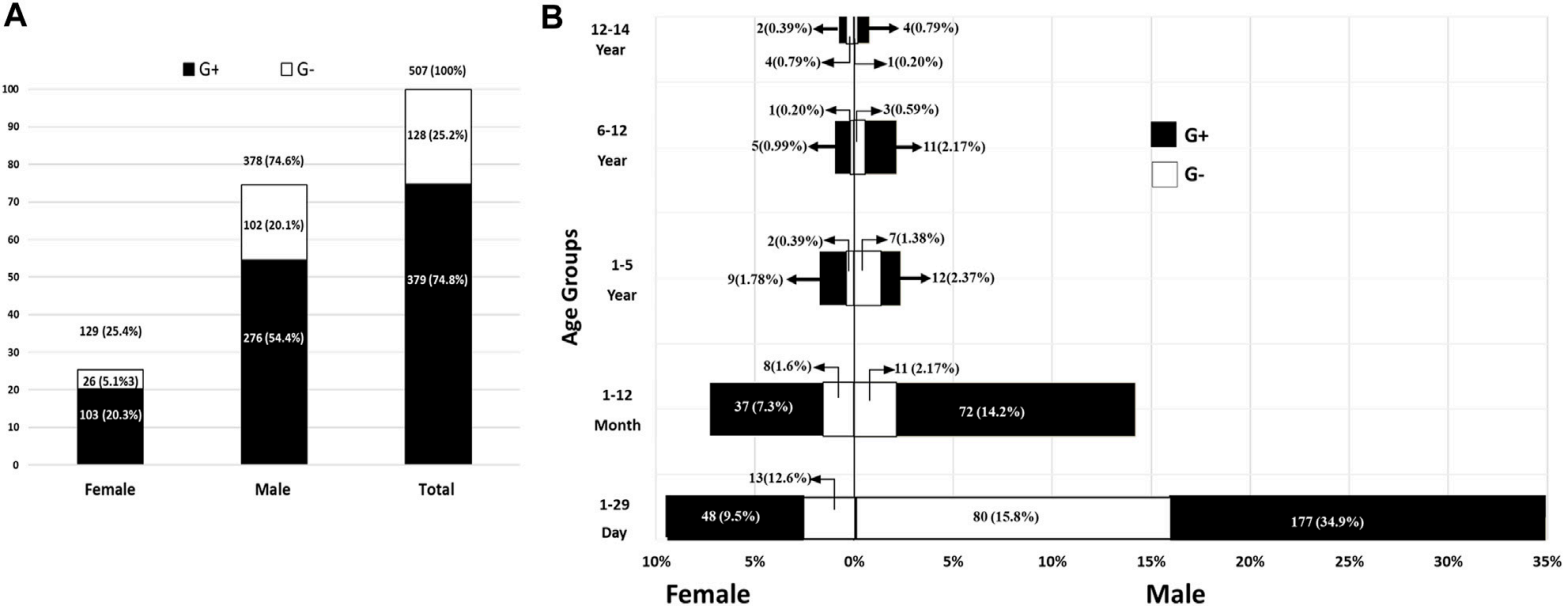
The overall count and rates of pediatric bacterial sepsis in both genders **(A)** and different age groups **(B)**. G+, Gram-positive bacteria; G-, Gram-negative bacteria.

In the current study, a total of 1,098 out- and in-patient children (<15 years) were recruited from between 1 January 2018, to 31 December 2021. Overall, 520 (47.35%) sepsis cases were found in 1,098 patients; 507 (97.5%) were caused by bacteria and 13 (2.5%) were caused by *Candida* (Supplementary Table S2), whereas no growth was observed in 578 (52.64%) of the 1,098 samples. The infection rate in males was 378 (74.6%), which was three-fold higher than that in females (129, 25.4%; Figure 1A). Moreover, the rate of Gram-positive bacterial infection was three-fold higher than Gram-negative bacterial infection (379 [74.75%] and 128 [25.25%], respectively). Most remarkably, for both sexes, the 1–29 days age group had the greatest prevalence of sepsis (28.96%), followed by the group up to 1 year (11.66%); the rates then gradually decreased until the age of 15 years (Figure 1B; Supplementary Table S2). Statistically, there was a significant difference between age group and the number of sepsis cases (*p* < 0.0001).

#### 3.1.1 Trends of bacterial infection

**FIGURE 2:**
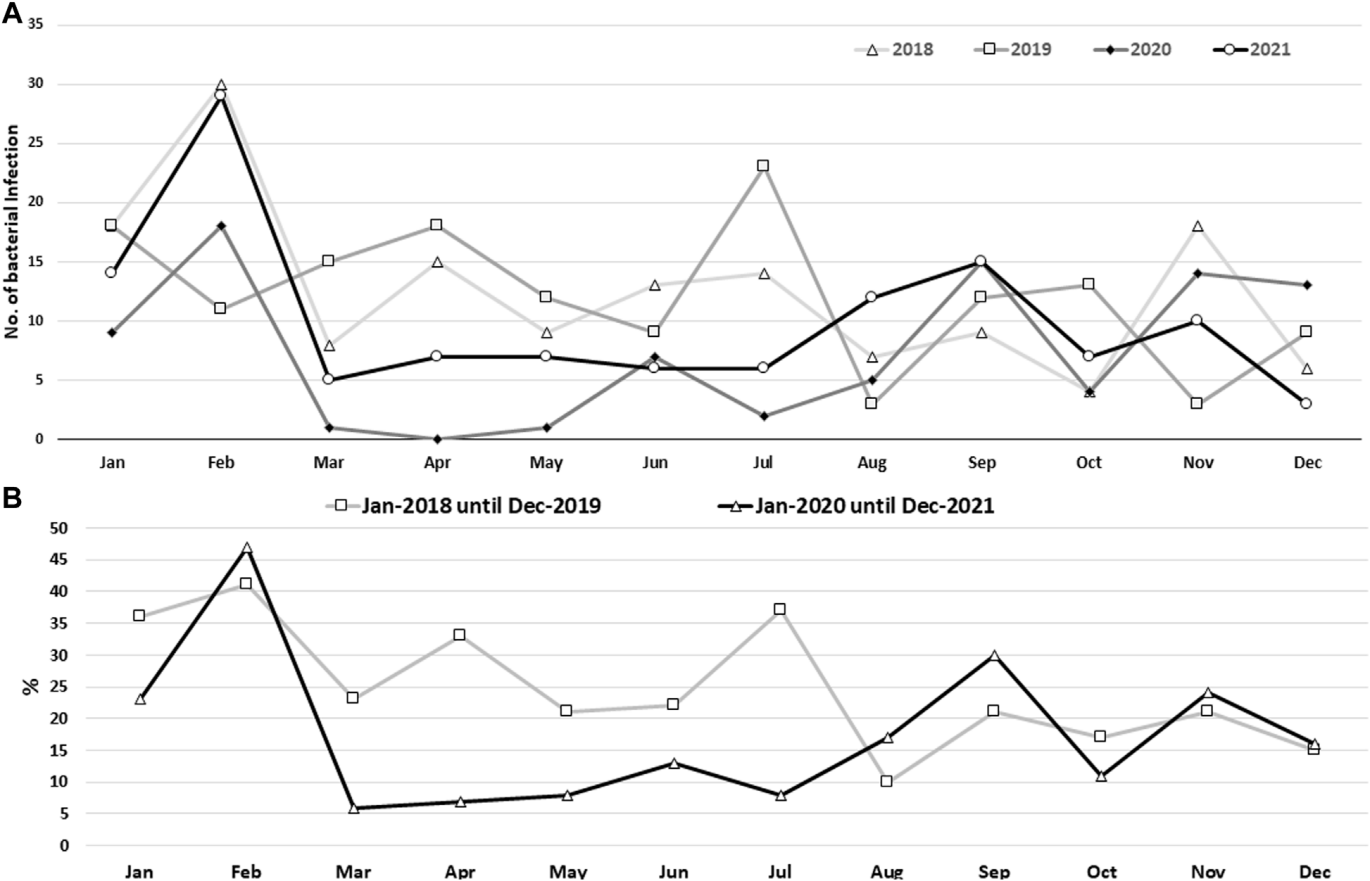
Bacterial sepsis rate based on monthly records **(A)** and the monthly record for phase I (pre-COVID-19 pandemic) and phase II (during the COVID-19 pandemic) **(B)** for the four-year data, 2018–2021. Using the Chi-square test, a statistically significant difference (*p* < 0.0001) was found between the various monthly data sets.

Overall, based on the monthly distribution of sepsis rates, the trend of sepsis reached a peak in February and decreased in the following months, with some fluctuation. However, in July 2021, the trend increased again (Figure 2A). Generally, a statistically significant difference (*p* < 0.0001) was observed between incidence rates based on monthly distribution using a chi-square test (Supplementary Table S3).

#### 3.1.2 Sepsis rate during the COVID-19 pandemic

As the main part of the study was conducted during the COVID-19 pandemic, a statistical analysis was conducted to compare the rates of sepsis during and before the pandemic. It had been noticed that the rate of sepsis was lower during the COVID-19 pandemic in 2020 and 2021 (since Kurdistan officially registered its first COVID-19 case in March 2020) than during the pre-pandemic period of 2018 and 2019, and according to a chi-square test the rates were significantly reduced (*p* < 0.0001; Figure 2B; Supplementary Table S3).

### 3.2 Bacterial causes of pediatric sepsis

The total number and percentages of various bacteria isolated from pediatric sepsis are shown in Table 1. Bacterial infection was higher in males than in females. Of the 507 strains cultured from children, 379 (74.75) were Gram positive and CoNS and methicillin-resistant *Staphylococcus aureus* (MRSA) were found in 264 (52.07%) and 45 (8.88%) cases, respectively. By contrast, *Klebsiella* spp. (55 cases, 10.85%) was the most common Gram-negative bacteria followed by non-lactose fermenter bacteria (42 cases, 8.28%) and *Escherichia coli* (29 cases, 5.72%) (Table 1). The main non-lactose fermenting bacteria identified were *Pseudomonas aeruginosa*, *Sphingomonas paucimobilis*, *Acinetobacter baumannii*, *Serratia* spp*.,* and *Stenotrophomonas maltophilia*.

**TABLE 1:** Sepsis-causing bacteria according to their phenotypic characteristics and based on the gender of patients.

Microorganism	Total No. (%)	Sex		*p*-value
		Female No. (%)	Male No. (%)	
G+ Bacteria	379 (74.75)	103 (20.32)	276 (54.44)	*p* = 0.0217
*Bacillus spp.*	7 (1.38)	—	7 (1.38)	
*Diphtheroids*	2 (0.39)	1 (0.2)	1 (0.2)	
*Enterococci*	27 (5.33)	11 (2.17)	16 (3.16)	
*Kocuria* spp.	4 (0.79)	1 (0.20)	3 (0.59)	
*Listeria monocytogenes*	1 (0.20)	—	1 (0.20)	
*Micrococcus luteus*	7 (1.38)	3 (0.59)	4 (0.79)	
*Methicillin-resistant Staphylococcus aureus*	45 (8.88)	13 (2.56)	32 (6.31)	
*Coagulase-negative Staphylococci (CoNS)*	264 (52.07)	68 (13.41)	196 (38.66)	
*Staphylococcus aureus*	18 (3.55)	6 (1.18)	12 (2.37)	
*Streptococci* spp.	4 (0.79)	—	4 (0.79)	
G- Bacteria	128 (25.25)	26 (5.13)	102 (20.12)	*p* = 0.2404
*Escherichia coli*	29 (5.72)	7 (1.38)	22 (4.34)	
*Klebsiella pneumonia* ^a^	55 (10.85)	8 (1.58)	47 (9.27)	
*Moraxella* spp.	1 (0.2)	1 (0.2)	—	
*Neisseria* spp.	1 (0.2)	—	1 (0.2)	
*Non-lactose fermenter* ^b^	42 (8.28)	10 (1.8)	32 (6.31)	

^a^ *Only 1 species K. oxytoca*.

^b^ Include all non-lactose fermenter bacteria such as *Pseudomonas aeruginosa, Sphingomonas paucimobilis, Acinetobacter baumannii, Serratia* spp*., and Stenotrophomonas maltophilia*.

### 3.3 Bacterial antibiotic resistance profile

**FIGURE 3:**
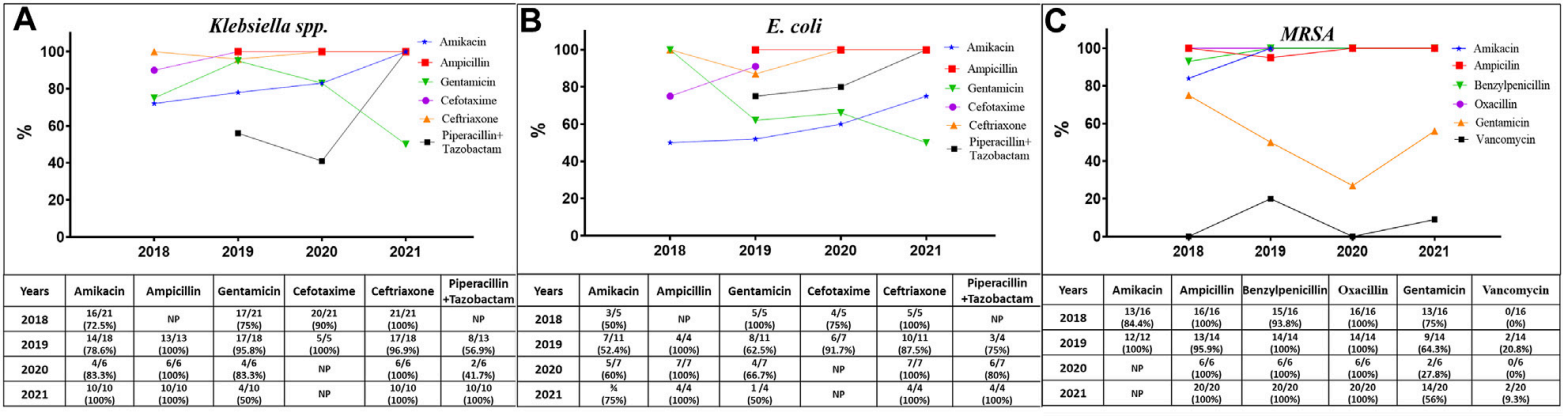
The trends of resistance of *Klebsiella* spp. **(A)**, *E. coli* **(B)**, and MRSA **(C)** to the antibiotics recommended by the World Health Organization for the treatment of pediatric sepsis. NP, not performed.

The antibiotic resistance profiles of the isolated bacteria were varied according to the time of the study and the type of bacteria. The World Health Organization (WHO) has recommended guidelines for the treatment of sepsis in neonates and children (WHO, 2022) (Supplementary Table S4). The WHO recommends two categories for treatment: access antibiotics (first choice) and watch antibiotics (second choice). Figure 3 illustrates the efficiency of the suggested antibiotics together with the resistance rate trends for G+ bacteria represented by *Staphylococcus aureus*, including MRSA, and G-bacteria represented by *Klebsiella pneumoniae* and *E. coli.* Supplementary Tables S5, S6 provide specifics about antibiotic resistance for these microorganisms.

In general, during the whole period of the study (Supplementary Table S5), all MRSA were resistant to AK, AMC, AMP, P, and OXI, while the resistance trend of these bacteria to GM was reduced from 75% (13/16) in 2018 to 64.3% and 27.8% in 2019 and 2020, respectively, and then increased again to 70% in 2021. Furthermore, the resistance values to vancomycin (VA) fluctuated across the 4-year study period, as the resistance rates were 0%, 21%, 0%, and 9%, respectively. In addition, all *Klebsiella* spp. isolates exhibited complete resistance to AMP, CTX, and CRO, while there was a variation in their resistant to AK, GM, and TZP. The resistant trends for AK and TZP were increased from above 50%–100% in 2021, whereas the trends against GM were almost resistant in the first 3 years and then decreased to 50% in 2021. In addition, all *E. coli* isolates were completely resistant to AMP and CRO, whereas their resistant rates to AK, GM, CTX, and TZP varied. The resistant trends increased from 50% to 75% for AK and from 75% to 100% for CTX and TZP, whereas the trend decreased from 100% resistance to 50% for GM in 2021.

In this investigation, MDR bacteria were prevalent. Some of the G+ and G-bacteria exhibited complete resistance to every class of antibiotic that was tested. Certain strains of *S. aureus* isolates, including MRSA, were also identified as MDR bacteria, showing sensitivity to only one antibiotic (vancomycin) within the tested antibiotics group. Some of the G-isolates were also 100% resistant to all the tested antibiotics, such as non-lactose fermenters, *Klebsiella* spp., and *E. coli*, while many other isolates demonstrated susceptibility to only one or two of the tested antibiotics, such as sulphamethaxazol/trimethoprim and imipenem.

### 3.4 Morbidity and mortality rates among pediatric sepsis patients

In terms of mortality rate, 47 (9.7%) out of 507 sepsis patients died. The mortality rate of pediatric sepsis caused by G+ bacteria was significantly higher than that caused by G- bacteria. The most common bacteria related to death in sepsis cases were CoNS (25 cases) and G-none-lactose fermenters (8 cases). Furthermore, sepsis brought on by MRSA (7 cases), *S. aureus* (3 cases), *E. coli* (2 cases), and *Klebsiella pneumoniae* (2 cases) resulted in an additional 14 deaths. It is noteworthy that all the deceased patients received local empirical treatment.

## 4 Discussion

In the current study, 46.17% of children suffered from sepsis because of bacterial infection; the incidence rate among males was three times greater than that of females. This finding contrasted with studies carried out in Austria by Hermon et al. (2021) and Libya by Benamer et al. (2015) that found that the rates of bacterial sepsis were comparable in both genders. In addition, a global study on the prevalence of sepsis showed that the rate of sepsis using age-standardized values is higher in females than in males (Rudd et al., 2020). The higher incidence of pediatric bacterial sepsis in males compared with females is likely due to a complex interplay of biological and environmental factors, proper hygiene, and immunization. Numerous studies have demonstrated sex-specific differences in sepsis and others infectious diseases. It has been found that females are protected under such conditions. However, a number of factors, such as a diminished cell-mediated immune response, cardiovascular functions, and some biological differences, especially the weight of X-linked variability and the role of sex hormones, can make males more vulnerable to such infection (De La Rica et al., 2016). However, it has been noted that despite reports of a higher prevalence of sepsis in males than in females, the exact mechanism underlying sepsis is still unknown (Xu et al., 2019).

The current study showed that the frequency of bacterial sepsis was more commonly observed in a subgroup of 1–29 days of age, followed by 1–12 months. It is thought that the maternal obstetric factors are the main cause of early-onset infections and sepsis in infants (Flannery et al., 2022). In fact, this might be due to the transfer of pathogenic bacteria to the fetus not only by crossing the placenta but also perinatally through the birth canal during delivery. Indeed, late-onset neonatal sepsis is caused by environmental factors, such as hospital and invasive procedures as well as the prolonged use of antibiotics (Rannikko et al., 2017; Herbozo et al., 2021).

Intriguingly, in the current study, the rate of sepsis was significantly different and decreased from 2018 to 2021. This finding was consistent with the global trend of sepsis, as the incidence of age-standardized sepsis decreased by 37·0% (95% uncertainty interval (UI)) from 1990 to 2017, which varied substantially across regions, with the highest burden in sub-Saharan Africa, Oceania, south Asia, east Asia, and southeast Asia (Rudd et al., 2020). By contrast, an epidemiological trends study of sepsis in western countries stated that the incidence of sepsis had increased over recent years (De La Rica et al., 2016). The increase in sepsis over time might be due to the aging population, an increase in comorbidities, and potential overuse of sepsis coding for less severe cases (Danai and Martin, 2005).

**FIGURE 4:**
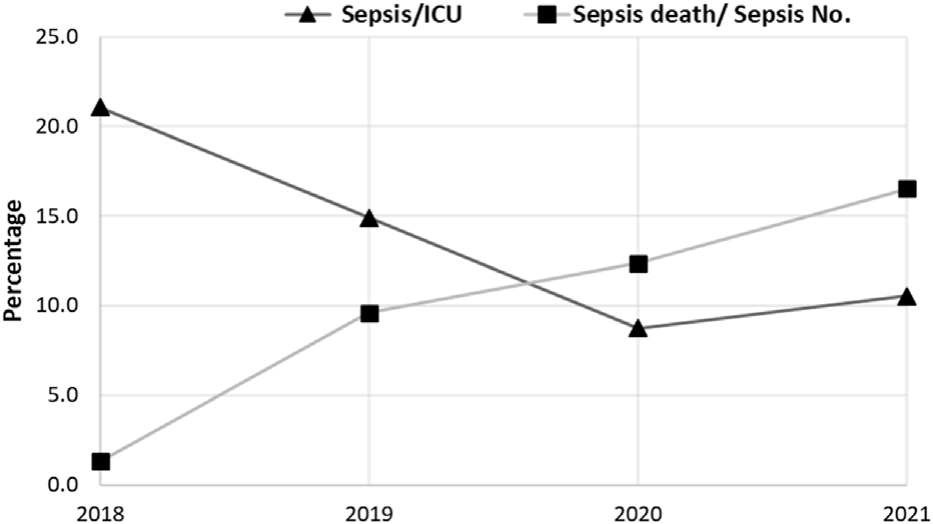
The rate of morbidity and mortality within sepsis cases in the intensive care unit (ICU) for the 4-year study (2018–2021). The prevalence of morbidity sepsis decreased from 154, 146, and 89 cases in 2018, 2019, and 2020, respectively, and then increased (121 cases) in 2021. The prevalence of mortality significantly increased from 1.3% (2/154 cases), 9.6% (14/46 cases), 12.4% (11/89 cases), and 16.5% (20/121 cases) in 2018, 2019, 2020, and 2021, respectively. The Chi-square test was used to evaluate the data, and significant differences (*p* < 0.0001) were observed.

In contrast to the rate of sepsis reduction during the current study period, the rate of mortality increased significantly during the same period, Figure 4. This was in line with the increasing rate of antibiotic resistance and the emergence of MDR bacteria within the causative agents of sepsis. The rate of mortality was higher in males than in females, which was expected as the rate of sepsis was higher in males than in females. This finding was consistent with another study that found a higher mortality rate in males (70%) than in females, which was related to the variation in the respiratory tract infection rate and IL-6 plasma levels between males and females (Nasir et al., 2015).

According to the monthly recorded data of the entire study period, the highest incidence of sepsis was recorded in February, except in 2019, when the highest incidence was in July. This finding was not surprising as previous studies recorded seasonal variation in sepsis rates, especially during winter (Benamer et al., 2015; Abdel Mawla et al., 2021). The incidence and death rate of sepsis were highest during winter, particularly for respiratory sepsis (respiratory tract infection), in which respiratory viral infection in sepsis patients played an important role in the progression of the infection (Aykac et al., 2019).

Compared with previous years, the COVID-19 pandemic had the lowest rate of sepsis. This could be because people sought care at outpatient clinics during the pandemic instead of staying in hospitals. Additionally, it might be due to reduced elective surgeries, changes in hospital admissions during the pandemic, such as for chronic conditions, and heightened infection control measures. It has been reported that one of the main reasons for the increased mortality due to sepsis during the COVID-19 pandemic was the reluctance of patients to seek medical attention or present themselves at a hospital (Unterberg et al., 2022).

With regard to the prevalence of sepsis-causing bacteria in the current study, CoNS and *Klebsiella pneumoniae* were the most predominate bacteria among G+ and G-bacteria, respectively. This was in line with a previous study conducted in a neonatal intensive care unit in Arab states in the Gulf region (Hammoud et al., 2017). By contrast, several previous studies reported that G-bacteria were the most common pathogens that led to sepsis (Danai and Martin, 2005; Hafsa et al., 2011; Flannery et al., 2022).

G+ and G-bacteria showed increasing resistance to antibiotics and the trend rates were characterized by fluctuations over time. This was due to several factors and mainly the normal evolutionary process of microorganisms, which is accelerated by the overuse and misuse of antibiotics, poor infection control, a lack of access to quality medicines, a lack of awareness and knowledge, and a lack of enforcement of legislation (Tessema et al., 2021).

Various studies observed that bacteria isolated from blood displayed a high level of resistance, not only to commonly used antibiotics but also to broad-spectrum antibiotics (Saleem et al., 2013; Ibrahim et al., 2023). To effectively control infections, the prolonged use of broad-spectrum antibiotics can lead to a resurgence of multidrug-resistant organisms; therefore, they should be used sparingly. Instead, specific short-term use of antibiotics is recommended, and the rotation of antibiotic regimens may also be a solution (Hammoud et al., 2017).

Furthermore, there is no universally accepted method for identifying and diagnosing sepsis and that delays in doing so can result in a significant increase in both morbidity and mortality (Rannikko et al., 2017; Oruganti et al., 2022).

This study has some limitations, including the manual methods employed for bacterial identification prior to the adoption of the VITEK system, which does not identify some bacteria at the species level, such as non-lactose fermenter bacteria. Additionally, it was unclear whether the infections were hospital-acquired or community-acquired, whether the patients had other infections, and whether they were taking medication prior to the sepsis infection. Furthermore, the survival status of patients after receiving antibiotics was unclear; the rate of deaths was stated according to the data reordered but there may have been additional occurrences if the patients were released from the hospital and then developed a new complication.

## 5 Conclusion

Our study evaluated the rate of sepsis in newborns and children in one hospital over the course of 4 years, from 2018 to 2021. The study period divided into 2 intervals: before and after the COVID-19 pandemic. In addition, the death rate and antibiotic resistance rate for the sepsis etiological agent were assessed in the study cases. Sepsis should be recognized quickly and managed promptly, otherwise it can lead to septic shock, multiple organ failure, and death. It is most frequently a serious complication of infection. In our study, many sepsis cases were identified and treated but then died, which might be due to treatment failure or organ failure but has not been properly investigated until now. Consequently, continuous efforts should be provided to reduce the burden of sepsis by improving public and healthcare worker awareness, especially to avoid delays in diagnosis and treatment. Furthermore, rapid treatment using appropriate antibiotics should be followed along with monitoring of the effectiveness of the treatment throughout the treatment period, especially with the increasing emergence of MDR bacteria within the isolated bacteria.

## Data Availability

The original contributions presented in the study are included in the article/Supplementary Material, further inquiries can be directed to the corresponding author.
