# Supplementary material for: Retrospective analysis of pediatric sepsis and the burden of antimicrobial resistance in Duhok, Kurdistan Region of Iraq
Source: Front Pharmacol. 2024 Feb 26;15:1347832. doi: 10.3389/fphar.2024.1347832 (PMC10925647; doi:10.3389/fphar.2024.1347832)
Supplement: Supplementary file 1 [file Table1.docx]

**Retrospective Analysis of Pediatric Sepsis and The Burden of Antimicrobial Resistance in Intensive Cares Units in Duhok, Kurdistan Region of Iraq**

Table S1. Demographic age groups among pediatric sepsis.

| Age Group | Age |
| --- | --- |
| New-born | 1-29 days |
| Infant | 1-12 Months |
| Toddler | 1-5 Years |
| School age | 6-11 Years |
| Adolescent | 12-14 Years |

Table S2. Frequency of *Candida* and bacteria identified from pediatric sepsis.

| **Age group** | **Bacterial infection**  **No.^*^ (%)** | ***Candida* infection**  **No. (%)** | **Non-growth**  **No. (%)** | **Total**  **No. (%)** |
| --- | --- | --- | --- | --- |
| 1-29 days | 318 (28.96) | 5 (0.46) | 244 (22.22) | 567 (51.64) |
| 1-12 months | 128 (11.66) | 6 (0.55) | 125 (11.38) | 259 (23.59) |
| 1-5 years | 30 (2.73) | 0 (0.00) | 138 (12.57) | 168 (15.30) |
| 6-12 years | 20 (1.82) | 1 (0.09) | 53 (4.83) | 74 (6.74) |
| 12-14 years | 11 (1.00) | 1 (0.09) | 18 (1.64) | 30 (2.73) |
| **Total No. (%)** | **507 (46.17)** | **13 (1.19)** | **578 (52.64)** | **1098 (100)** |

Statistically, there was a significant difference between the age group and the number of infections (p<0.0001).

Table S3. Number of monthly bacterial infections throughout a 4-year period, including before and after the COVID-19 pandemic.

|  | 4 years of study | | | | Pre-pandemic period | COVID-19 pandemic period |
| --- | --- | --- | --- | --- | --- | --- |
| Months* | 2018 | 2019 | 2020 | 2021 | January 2018- December 2019 | January 2020- December 2021 |
| Jan | 18 | 18 | 9 | 14 | 36 | 23 |
| Feb | 30 | 11 | 18 | 29 | 41 | 47 |
| Mar | 8 | 15 | 1 | 5 | 23 | 6 |
| Apr | 15 | 18 | 0 | 7 | 33 | 7 |
| May | 9 | 12 | 1 | 7 | 21 | 8 |
| Jun | 13 | 9 | 7 | 6 | 22 | 13 |
| Jul | 14 | 23 | 2 | 6 | 37 | 8 |
| Aug | 7 | 3 | 5 | 12 | 10 | 17 |
| Sep | 9 | 12 | 15 | 15 | 21 | 30 |
| Oct | 4 | 13 | 4 | 7 | 17 | 11 |
| Nov | 18 | 3 | 14 | 10 | 21 | 24 |
| Dec | 6 | 9 | 13 | 3 | 15 | 16 |

*Statistical analysis performed using the chi-square test found a significant difference (p<0.0001) between monthly counts of bacterial infections. A significant difference (p<0.0001) was additionally found between the data collected before and after the COVID-19 pandemic.

Table S4. WHO empirical treatment recommendation for hospitalized children with sepsis.

| **First choice** | |
| --- | --- |
| Antibiotics | Access/watch |
| Ampicillin or benzylpenicillin combined with gentamicin | Access  Access |
| **Second Choice** | |
| Cefotaxime or ceftriaxone  Or cloxacillin  combined with  amikacin | Watch  Access  Access |
| Piperacillin tazobactam^*^ | Watch |

* The list of antibiotics that have activity against Gram-negative bacteria and can be used if a high prevalence of resistance is suspected, particularly healthcare-associated infections.

Table S5. Antibiotic susceptibility among MRSA^*^ and *S.* *aureus*.

| **Antibiotic classification** | **Penicillins** | | | | | | | | **Aminoglycosides** | | | | **Cephalosporin (1st generation)** | | | **Cephalosporins (2nd generation)** | | **Fluoroquinolones** | | **Fusidane** | | **Glycopeptides** | | | | **lipopeptide** | | **Macrolides** | | | | | | **Oxazolidinones** | | **Phosphonic** | | **Sulfonamides** | | **Tetracyclines** | | | |
| --- | --- | --- | --- | --- | --- | --- | --- | --- | --- | --- | --- | --- | --- | --- | --- | --- | --- | --- | --- | --- | --- | --- | --- | --- | --- | --- | --- | --- | --- | --- | --- | --- | --- | --- | --- | --- | --- | --- | --- | --- | --- | --- | --- |
| **Antibiotic disc** | **Amoxiclav (AMC)** | | **Ampicillin (AMP)** | | **Benzylpenicillin**  **(P)** | | **Oxacillin (OXI)** | | **Amikacin**  **(AK)** | | **Gentamycin (GM)** | | | **Cephalothin (Kf)** | | **Cefoxitin (OXSF)** | | **Ciprofloxacin (CIP)** | | **Fusidic acid (FA)** | | **Teicoplanin (TEC)** | | **Vancomycin**  **(VA)** | | **Daptomycin (DAP)** | | **Azithromycin (Azm)** | | **Clindamycin (Da)** | | **Erythromycin (E)** | | **Linezolid (LNZ)** | | **Fosfomycin (FOS)** | | **Trimethoprim/ Sulfamethoxazole (SXT)** | | **Tetracycline (TE)** | | **Tigecycline**  **(TGC)** | |
| G+ bacteria^***^ | R/N**  (%) | S/N  (%) | R/N  (%) | S/N  (%) | R/N  (%) | S/N  (%) | R/TN  (%) | S/N  (%) | R/N  (%) | S/N  (%) | R/N  (%) | S/N  (%) | | R/N  (%) | S/N  (%) | R/N  (%) | S/N  (%) | R/N  (%) | S/N  (%) | R/N  (%) | S/N  (%) | R/N  (%) | S/N  (%) | R/N  (%) | S/N  (%) | R/N  (%) | S/N  (%) | R/N  (%) | S/N  (%) | R/N  (%) | S/N  (%) | R/N  (%) | S/N  (%) | R/N  (%) | S/N  (%) | R/N  (%) | S/N  (%) | R/N  (%) | S/N  (%) | R/N  (%) | S/N  (%) | R/N  (%) | S/N  (%) |
| *MRSA* | 27/27  (100) | 0 | 44/45  (97.78) | 1/45  (2.22) | 44/45  (97.78) | 1/45  (2.22) | 45/45  (100) | 0 | 24/27  (88.89) | 3/27  (11.11) | 33/45  (73.33) | 12/45  (26.67) | | 26/27  (96.30) | 1/27  (3.70) | 18/18  (100) | 0 | 18/18  (100) | 0 | 16/18  (88.89) | 2/18  (11.11) | 17/18  (94.44) | 1/18  (5.56) | 1/45  (2.22) | 44/45  (97.78) | 17/18  (94.44) | 1/18  (5.56) | 25/27  (92.59) | 2/27  (7.41) | 11/45  (24.44) | 34/45  (75.56) | 44/45  (97.78) | 1/45  (2.22) | 16/18  (88.89) | 2/18  (11.11) | 18/18  (100) | 0 | 11/18  (61.11) | 7/18  (38.89) | 18/18  (100) | 0 | 16/18  (88.89) | 2/18  (11.11) |
| *Staphylococcus aureus* | 4/4  (100) | 0 | 18/18  (100) | 0 | 18/18  (100) | 0 | 11/18  (61.11) | 7/18  (38.89) | 3/4  (75) | 1/4  (25) | 8/18  (44.44) | 10/18  (55.56) | | 0 | 4/4  (100) | 14/14  (100) | 0 | 14/14  (100) | 0 | 12/14  (85.71) | 2/14  (14.29) | 12/14  (85.71) | 2/14  (14.29) | 3/18  (16.67) | 15/18  (83.33) | 13/14  (92.86) | 1/14  (7.14) | 2/4  (50) | 2/4  (50) | 4/18  (22.22) | 14/18  (77.78) | 17/18  (94.44) | 1/18  (5.56) | 12/14  (85.71) | 2/14  (14.29) | 12/14  (85.71) | 2/14  (14.29) | 10/14  (71.43) | 4/14  (28.57) | 12/14  (85.71) | 2/14  (14.29) | 12/14  (85.71) | 2/14  (14.29) |

^*^MRSA, Methicillin-resistant *Staphylococcus aureus*; ^**^N, total number; ^***^ G+, Gram positive

Table S6. Antibiotic susceptibility among *E. coli* and *Klebsiella spp*.

| **Antibiotic classification** | Penicillins | | | | Aminoglycosides | | | | Carbapenems | | | | | | Cephalosporin (1st generation) | | Cephalosporins (2nd generation) | | | | | (3rd generation) | | | | | | | | | Fluoroquinolones | | Macrolides | | | | Nitrofurans | | Phosphonic | |  | | Sulfonamides | | Trimethoprim | |
| --- | --- | --- | --- | --- | --- | --- | --- | --- | --- | --- | --- | --- | --- | --- | --- | --- | --- | --- | --- | --- | --- | --- | --- | --- | --- | --- | --- | --- | --- | --- | --- | --- | --- | --- | --- | --- | --- | --- | --- | --- | --- | --- | --- | --- | --- | --- |
| **Antibiotic disc** | Amoxiclav (AMC) | | Ampicillin (AMP) | | Amikacin  (AK) | | Gentamycin (GM) | | Ertapenem (ETP) | | Imipenem  (IPM) | | Meropenem (MEM) | | Cephalothin (Kf) | | Cefoxitin (OXSF) | | Cefuroxime (CXM) | | Cefixime (CFM) | | | Cefotaxime (Ctx) | | Ceftazidime (CAZ) | | Ceftriaxone (CRO) | | Ciprofloxacin (CIP) | | | Azithromycin (Azm) | | Clindamycin (Da) | | Nitrofurantoin (FT) | | Fosfomycin (FOS) | | Piperacillin/ Tazobactam (TZP) | | Trimethoprim/ Sulfamethoxazole (SXT) | | Trimethoprim (Tmp) | |
| **G – bacteria^**^** | R/N^*^  (%) | S/N  (%) | R/N  (%) | S/N  (%) | R/N  (%) | S/N  (%) | R/N  (%) | S/N  (%) | R/N  (%) | S/N  (%) | R/N  (%) | S/N  (%) | R/N  (%) | S/N  (%) | R/N  (%) | S/N  (%) | R/N  (%) | S/N  (%) | R/N  (%) | S/N  (%) | R/N  (%) | | S/N  (%) | R/N  (%) | S/N  (%) | R/N  (%) | S/N  (%) | R/N  (%) | S/N  (%) | R/N  (%) | | S/N  (%) | R/N  (%) | S/N  (%) | R/N  (%) | S/N  (%) | R/N  (%) | S/N  (%) | R/N  (%) | S/N  (%) | R/N  (%) | S/N  (%) | R/N  (%) | S/N  (%) | R/N  (%) | S/N  (%) |
| ***E. coli*** | 26/29  (89.66) | 3/29  (10.34) | 17/17  (100) | 0 | 20/29  (68.97) | 9/29  (31.03) | 20/29  (68.97) | 9/29  (31.03) | 16/17  (94.12) | 1/17  (5.88) | 7/29  (24.14) | 22/29  (75.86) | 25/29  (86.21) | 4/29  (13.79) | 12/12  (100) | 0 | 16/17  (94.12) | 1/17  (5.88) | 16/17  (94.12) | 1/17  (5.88) | 27/29  (93.10) | | 2/29  (6.90) | 10/12  (83.33) | 2/12  (16.67) | 17/17  (100) | 0 | 28/29  (96.55) | 1/29  (3.45) | 14/17  (82.35) | | 3/17  (17.65) | 12/12  (100) | 0 | 12/12  (100) | 0 | 16/17  (94.12) | 1/17  (5.88) | 15/17  (88.24) | 2/17  (11.76) | 15/17  (88.24) | 2/17  (11.76) | 7/17  (41.18) | 10/17  (58.82) | 6/12  (50) | 6/12  (50) |
| ***Klebsiella spp.*** | 53/55  (96.36) | 2/55  (3.64) | 29/29  (100) | 0 | 44/55  (80) | 11/55  (20) | 42/55  (76.36) | 13/55  (23.64) | 24/29  (82.76) | 5/29  (17.24) | 15/55  (27.27) | 40/55  (72.73) | 44/55  (80) | 11/55  (20) | 26/26  (100) | 0 | 27/29  (93.10) | 2/29  (6.90) | 29/29  (100) | 0 | 53/55  (96.36) | | 2/55  (3.64) | 25/26  (96.15) | 1/26  (3.85) | 28/29  (96.55) | 1/29  (3.45) | 54/55  (98.18) | 1/55  (1.82) | 20/29  (68.97) | | 9/29  (31.03) | 26/26  (100) | 0 | 26/26  (100) | 0 | 27/29  (93.10) | 2/29  (6.90) | 24/29  (82.76) | 5/29  (17.24) | 20/29  (68.97) | 9/29  (31.03) | 7/29  (24.14) | 22/29  (75.86) | 22/26  (84.62) | 4/26  (15.38) |

^*^N, total number; ^**^ G-, Gram negative

Table S7. The total number and the rate of morbidity and mortality within sepsis cases in intensive care units from 2018 to 2021.

|  | 2018 | 2019 | 2020 | 2021 |
| --- | --- | --- | --- | --- |
|  | Number or % | Number or % | Number or % | Number or % |
| **Intensive care total number** | 717 | 980 | 1019 | 1149 |
| **Sepsis total number** | 154 | 146 | 89 | 121 |
| **% of sepsis No./intensive care total number** | 21.1 | 14.9 | 8.7 | 10.5 |
| **Sepsis deaths total number** | 2 | 14 | 11 | 20 |
| **% of total deaths/sepsis total number** | 1.3 | 9.6 | 12.4 | 16.5 |
